# Comparison of Stable and Transient *Wolbachia* Infection Models in *Aedes aegypti* to Block Dengue and West Nile Viruses

**DOI:** 10.1371/journal.pntd.0005275

**Published:** 2017-01-04

**Authors:** Dirk Albert Joubert, Scott L. O’Neill

**Affiliations:** Institute of Vector-borne Disease, Monash University, Clayton, Australia; University of Tennessee, UNITED STATES

## Abstract

Pathogen replication and transmission in *Wolbachia* infected insects are currently studied using three *Wolbachia* infection systems: naturally infected *Wolbachia* hosts, hosts transinfected with *Wolbachia* (stably maintained and inherited infections) and hosts transiently infected with *Wolbachia*. All three systems have been used to test the effect of *Wolbachia* on mosquito transmitted pathogens such as dengue virus (DENV), West Nile virus (WNV) and *Plasmodium*. From these studies it is becoming increasingly clear that the interaction between a particular pathogen and *Wolbachia* is heavily influenced by the host-*Wolbachia* interaction and the model of infection. In particular, there is some evidence that under very specific conditions, *Wolbachia* can enhance pathogen infection in some hosts. In this study, we compared the effect of *Wolbachia* in two infection models (stable transinfected and transiently infected) on the replication, infection- and transmission rates of two flaviviruses, DENV and WNV (Kunjin strain). Our results indicate that *Wolbachia* had similar blocking effects in both stable and transient models of infection, however, the magnitude of the blocking effect was significantly lower in mosquitoes transiently infected with *Wolbachia*. More importantly, no evidence was found for any enhancement of either DENV or WNV (Kunjin strain) infection in *Ae*. *aegypti* infected with *Wolbachia*, supporting a role for *Wolbachia* as an effective and safe means for restricting transmission of these viruses.

## Author Summary

*Wolbachia* is a naturally occurring endosymbiotic bacterium that, when introduced into a naïve mosquito host, has been shown to effectively reduce the replication and transmission of pathogens such as dengue virus, West Nile virus, Chikungunya virus, yellow fever virus and *Plasmodium*. However, a recent study has indicated that, under certain conditions, transiently infected *Wolbachia* can enhance West Nile virus infection in *Culex tarsalis* mosquitoes. We wanted to investigate whether this enhancement effect could also be observed in *Aedes aegypti* mosquitoes and if so, whether it is specific to the nature of the *Wolbachia* infection model under study (transient vs stable). We compared the replication and transmission of dengue virus and WNV (Kunjin strain) in *Aedes aegypti* mosquitoes transiently infected with *Wolbachia* and mosquitoes stably infected with the identical *Wolbachia* strain. Contrary to the previous study, our results show no enhancement of replication or transmission for either dengue virus or WNV (Kunjin strain) in mosquitoes transiently or stably infected with *Wolbachia*.

## Introduction

The potential for *Wolbachia* as a natural control method for mosquito-borne pathogens such as dengue virus (DENV), Chikungunya virus (CHIKV), West Nile virus (WNV), yellow fever virus (YFV) and malaria has been the focus of intense study in recent years [1–8]. The majority of these studies have focussed on DENV replication and have shown conclusively that *Wolbachia* effectively reduces DENV replication and transmission when introduced as a stable infection in the naturally uninfected host *Aedes aegypti* [8–11].

To date, only a few mosquito species have been successfully transinfected with *Wolbachia*. These include *Ae*. *aegypti* (transinfected with the *Wolbachia* strains *w*Mel, *w*MelPop, *w*AlbB, and superinfection with *w*Mel*w*AlbB [6, 8–10]), *Ae*. *albopictus* (cured of its natural *Wolbachia* infection and transinfected with the *w*Mel *Wolbachia* strain [12]), and *Ae*. *polynesienses* and *Anopheles stephensi* (both transinfected with the *w*AlbB *Wolbachia* strain [13, 14]). These transinfected strains have shown excellent potential for the biocontrol of several important mosquito-transmitted diseases (for recent reviews see [15–17]). However, several disease transmitting mosquito species remain recalcitrant to *Wolbachia* transinfection, hampering efforts to better understand the interaction between *Wolbachia*, it’s host and disease causing pathogens [18].

Natural *Wolbachia* infection models have therefore also been examined to provide insight into *Wolbachia*-host-pathogen interactions. In this model, the naturally occurring *Wolbachia* infection is first cured from the host and pathogen replication is subsequently compared in cured and naturally infected hosts [19, 20].

Using this model Baton et al. found that *w*Flu infection in its natural host *Ae*. *fluviatilis*, enhanced oocyst infection with the avian malaria parasite *P*. *gallinaceum* [19, 20]. Zele et al. also showed that in the natural mosquito-*Wolbachia*-*Plasmodium* combination, *Wolbachia* increased the susceptibility of *Culex pipiens* mosquitoes to *P*. *relictum* [20]. Furthermore, a study by Mousson et al. using this model, found that *Ae*. *albopictus* naturally superinfected with the two *Wolbachia* strains (*w*AlbA and *w*AlbB) infection limited the transmission, but not replication of DENV. Here, both the naturally occurring *Wolbachia* strains were cured and the vector competence for DENV of the resulting uninfected line was compared to the superinfected line [21].

In addition to natural infection systems, transient infection systems have been used to investigate the effect of *Wolbachia* on *Plasmodium* and WNV infection. Here, *Wolbachia* is injected into an uninfected mosquito host and allowed to establish a transient somatic infection [22]. Using this model, the effect of the *Wolbachia* strains *w*AlbB and *w*MelPop on the malaria parasite *P*. *berghei* in *A*. *gambiae* was investigated [23]. Contrary to the results of [14], in this experimental setup, *w*AlbB was found to enhance *P*. *berghei* infection, whilst *w*MelPop only had a moderate blocking effect [23]. A more recent study utilised the same infection model to investigate the effect of the *w*AlbB *Wolbachia* strain on WNV infection in *Culex tarsalis* [24]. Contrary to previous studies that found *w*AlbB inhibited WNV infection in *Ae*. *aegypti* [5], *C*. *tarsalis* transiently infected with *w*AlbB enhanced WNV infection rates at 7 days post infection [24].

Together these results suggest that the degree of pathogen modulation from different host-*Wolbachia* combinations can differ considerably depending on the mode of infection, the host and the pathogen. Consequently, it is important not to base predictions of pathogen modulation in a particular host-*Wolbachia* strain combination on results obtained from divergent infection modes and host species. In this study we have compared the effect of *w*AlbB on replication and transmission of DENV and WNV (Kunjin strain) in *Ae*. *aegypti* infected through both transient somatic infection and stable transinfection. Our results showed significantly lower *Wolbachia* infection densities in transiently infected *Ae*. *aegypti* when compared to the stable infected line. More importantly, both *Wolbachia* infection models displayed similar effects, blocking replication and transmission of both DENV and WNV (Kunjin strain). These results conclusively show that neither DENV nor WNV (Kunjin strain) infection is enhanced in *Ae*. *aegypti* either transiently or stably infected with *w*AlbB.

## Results and Discussion

### *Wolbachia* density and distribution in transiently infected *Ae*. *aegypti* mosquitoes

**Fig 1:**
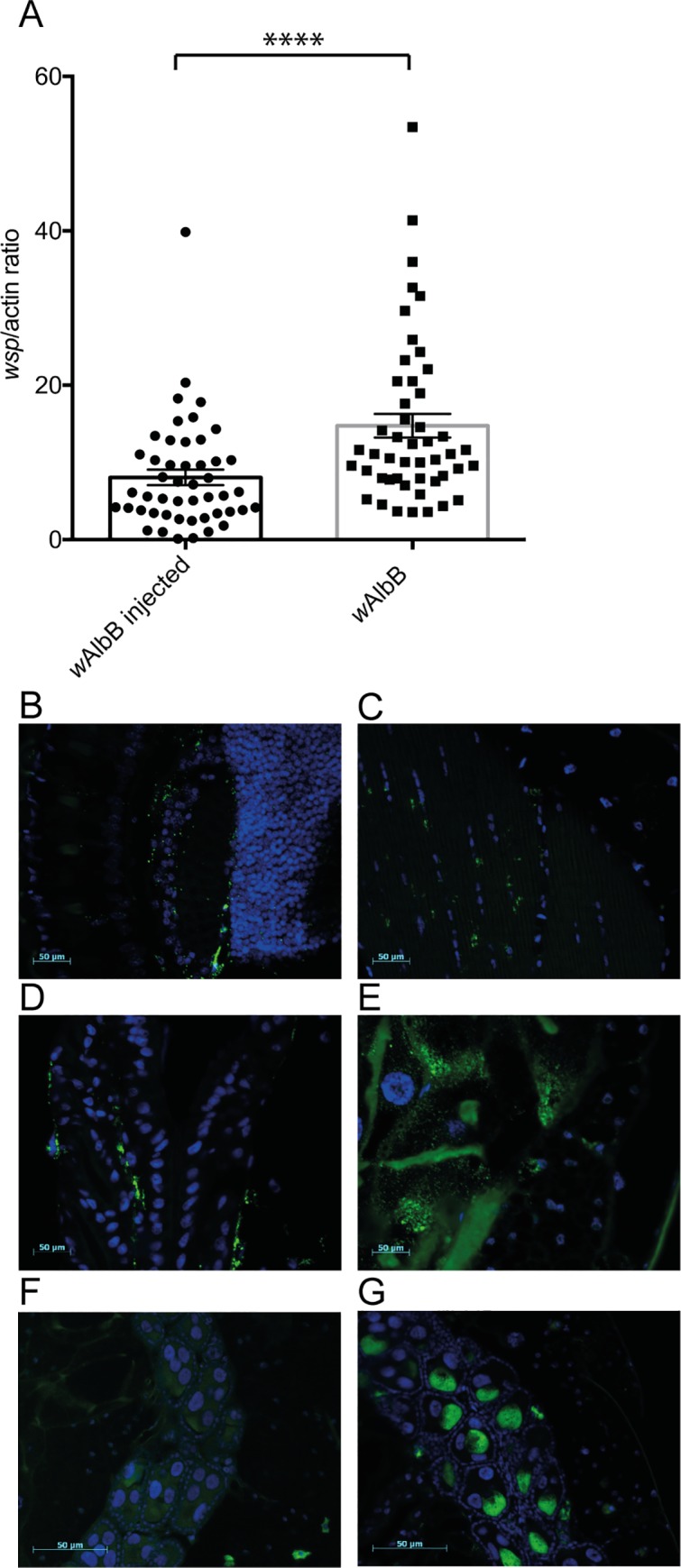
*Wolbachia* density and localisation in transiently infected *Aedes aegypti* 7 days post injection (dpi). A) The *wsp*/*actin* ratio in transiently infected *Ae*. *aegypti* compared to the stable transinfected strain. The combined results from two independent experiments are shown. *Wolbachia* density was determined using qPCR as described and the mean and error of the mean are indicated. Statistical significance was determined using a Mann-Whitney test (****, p < 0.0001). B-F) FISH staining of *w*AlbB (green) in transiently infected female mosquitoes. B) brain tissue, C) muscle tissue, D) midgut tissue, E) fat body, F) ovaries. G) *w*AlbB localisation in ovaries of the stable, transinfected line.

*Wolbachia* density and distribution was analysed in female *Ae*. *aegypti* mosquitoes transiently infected with the *w*AlbB *Wolbachia* strain and compared to the stable infected *w*AlbB line. *Wolbachia* density was determined using qPCR and primers specific to the *Wolbachia* surface protein (*wsp*) in conjunction with the *Ae*. *aegypti* actin gene for normalisation. In our experiments, even when *Wolbachia* was injected at very high densities (~10^11^ bacteria/mL), there were significantly lower (Mann-Whitney test, p = 0.007) *Wolbachia* densities at 7 days post injection (dpi) in the transiently infected mosquitoes than densities observed in the stable *w*AlbB infected line (Fig 1A).

*Wolbachia* in transiently infected mosquitoes were predominantly located in the brain (Fig 1B), muscle tissue (Fig 1C), the midgut (Fig 1D) and the fat body (Fig 1E). In stark contrast to the stable *w*AlbB infected line, however, very little to no *Wolbachia* could be detected in the ovaries of transiently infected lines (Fig 1F & 1G). In the stable *w*AlbB infected line, the vast majority of *Wolbachia* are found in the ovaries and the lack of *Wolbachia* found in the ovaries of transiently infected mosquitoes could explain the significant differences found in *Wolbachia* density between stable and transiently infected mosquitoes in our qPCR results (Fig 1A). These results are also consistent with previous studies that showed only limited *Wolbachia* localisation in the ovaries of transiently infected *Culex tarsalis* [24] and comparatively low levels of *Wolbachia* in the ovaries of transiently infected *Anopheles gambiae* compared to the rest of the body [25].

### DENV replication and transmission is reduced in female *Ae*. *aegypti* transiently infected with *w*AlbB

**Fig 2:**
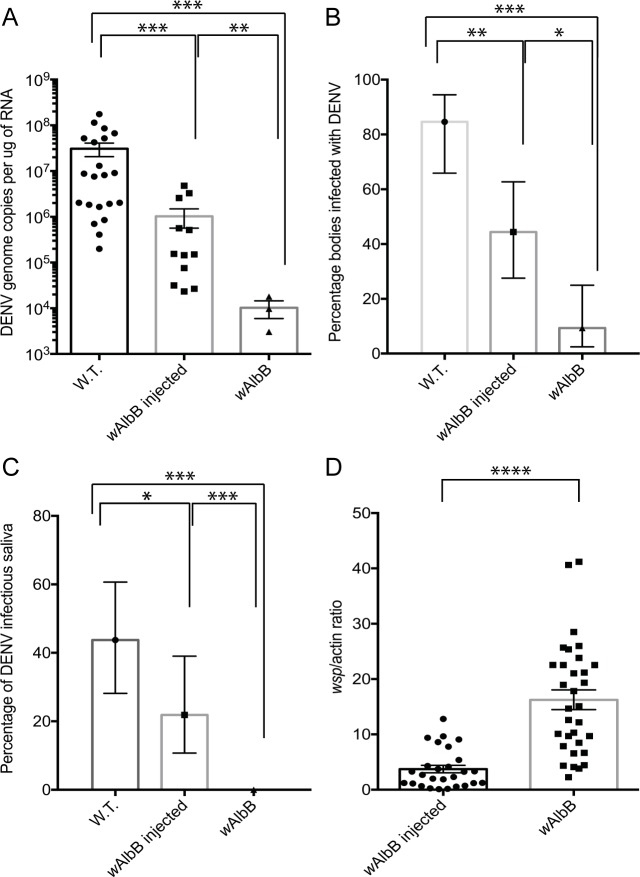
DENV infection, replication and transmission in wild type (W.T.), transiently infected (*w*AlbB injected) and the stable transinfected (*w*AlbB) lines 7 days post an infectious blood meal. The combined results of two independent experiments are shown (the results of each individual repeat are available as supplemental data in S2 Fig and S4 Fig). Statistical significance was determined using a Mann-Whitney test (A and D) or a Fisher exact test (B and C). In A and D the mean and error of the mean is indicated. In B and C, the error bars represent 95% confidence levels. A) DENV genome copies in whole mosquito bodies (***, p < 0.001; **, p = 0.004; Mann-Whitney). B) DENV infection rate as determined by the percentage of individuals infected 7 days post an infectious blood meal, n = 28 (***, p = 0.001; **, p = 0.004; *, p = 0.04; Fisher exact test). C) DENV transmission rate as determined by the percentage of infectious saliva expectorated 7 days post an infectious blood meal, n = 28 (***, p < 0.0004; *, p = 0.03; Fisher exact test). D) *Wolbachia* density 7 days post an infectious blood meal in transiently infected and the stable transinfected line (****, p < 0.0001; Mann-Whitney).

We next investigated whether female *Ae*. *aegypti* mosquitoes transiently infected with *w*AlbB displayed the same DENV blocking phenotype as the stable infected *w*AlbB line [24]. Townsville wild type (W.T.), W.T. transiently infected with *w*AlbB and stable *w*AlbB infected females were provided with a DENV infected blood meal 7 dpi. The mosquitoes were incubated for a further 7 days as described in materials and methods and subsequently analysed for DENV replication (Fig 2A), DENV infection rate (Fig 2B), DENV transmission rate (Fig 2C), as well as *w*AlbB density (Fig 2D).

DENV copy number (as determined by positive strand genome copy number) in the bodies of transiently infected females was significantly reduced (Mann-Whitney, p = 0.0002) by ~1.5 logs when compared to DENV replication in W.T. mosquitoes. *Ae*. *aegypti* stably infected with *w*AlbB showed the greatest reduction in + strand DENV genome copies with a ~ 3 log reduction compared to W.T. (Mann-Whitney, p = 0.001) and ~ 2 log reduction compared to transiently infected females (Mann-Whitney, p = 0.004). Similarly, the DENV infection rate was significantly reduced (~2 fold, Fisher exact test, p = 0.004) in *Ae*. *aegypti* females transiently infected with *w*AlbB when compared with W.T. females (Fig 2B). *Ae*. *aegypti* stably infected with *w*AlbB again showed the greatest reduction in DENV transmission rates with an ~8 fold reduction compared to W.T. females (Fisher exact test, p = 0.0001) and ~4 fold reduction compared to *Ae*. *aegypti* females transiently infected with *w*AlbB (Fisher exact test, p = 0.04).

DENV transmission in transiently infected *w*AlbB mosquitoes was significantly reduced compared to W.T. mosquitoes. Saliva was collected 7 days post feeding from females fed with an infected blood meal and then injected into DENV-naïve W.T. females according to [26]. The mosquitoes were incubated for an additional 7 days before analysing DENV infection status by qRT-PCR (Fig 2C). No DENV infectious saliva was detected from female *Ae*. *aegypti* mosquitoes stably infected with *w*AlbB. In contrast, 22% of female *Ae*. *aegypti* mosquitoes transiently infected with *w*AlbB expectorated DENV infectious saliva.

Finally, we analysed the *Wolbachia* density in the same transiently and stably infected mosquitoes analysed for DENV replication and transmission. Our results indicate much lower *Wolbachia* densities in the transiently infected females compared to the stable *w*AlbB infected line (Fig 2D). *Wolbachia* density has been correlated with the degree of pathogen blocking in *Wolbachia* infected hosts [27] and the lower densities in transiently infected mosquitoes observed here provides a plausible explanation for the reduced DENV blocking phenotype we observed in the transiently infected mosquitoes compared to the stable infected line.

### WNV (Kunjin strain) replication is reduced in female *Ae*. *aegypti* transiently infected with *w*AlbB

**Fig 3:**
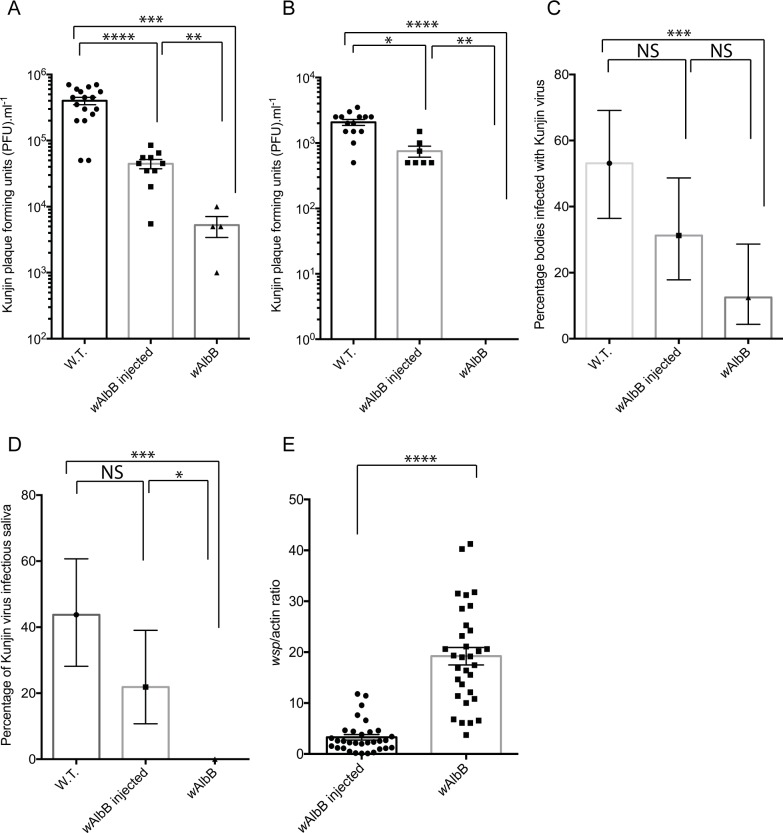
WNV (Kunjin strain) infection, replication and transmission in wild type (W.T.), transiently infected (*w*AlbB injected) and the stable transinfected (*w*AlbB) lines 7 days post an infectious blood meal. The combined results of two independent experiments are shown (the results of each individual repeat are available as supplemental data in S3 Fig and S5 Fig). Statistical significance was determined using a Mann-Whitney test (A, B and E) or a Fisher exact test (C and D). In A, B and E, the mean and error of the mean is indicated. In C and D, the error bars represent 95% confidence levels. A) WNV (Kunjin strain) PFU per ml in whole mosquito bodies, (****, p < 0.0001; ***, p = 0.0003, **, p = 0.003; Mann-Whitney). B) WNV (Kunjin strain) PFU per ml in saliva (****, p < 0.0001; **, p = 0.001; *, p = 0.01; Mann-Whitney). C) WNV (Kunjin strain) infection rate as determined by the percentage of individuals infected 7 days post an infectious blood meal, n = 32 (***, p = 0.001; Fisher exact test). D) WNV (Kunjin strain) transmission rate as determined by the percentage of infectious saliva expectorated infected 7 days post an infectious blood meal, n = 32 (*, p = 0.03; Fisher exact test). E) *Wolbachia* density 7 days post an infectious blood meal in transiently infected and the stable transinfected lines (****, p < 0.0001; Mann-Whitney).

We repeated the infection experiments using WNV (Kunjin strain). W.T. and *w*AlbB infected females were provided with a virus infected blood meal at 7 dpi. The mosquitoes were incubated for a further 7 days as described and virus titres were determined in whole bodies (Fig 3A) and saliva (Fig 3B). We also compared the virus infection (Fig 3C) and transmission rates (Fig 3D) between *Wolbachia* infected and uninfected mosquitoes.

Virus titres were significantly reduced in the bodies of both the transient and stable infected mosquito lines compared to W.T. females. Transient *Wolbachia* infection resulted in an approximate one log reduction (Mann-Whitney, p > 0.0001) in virus PFU (Fig 3A) in whole mosquito bodies. A small but significant (Mann-Whitney, p = 0.001), 0.28 log reduction in virus PFU was observed in saliva from these mosquitoes compared to W.T. (Fig 3B). The greatest reduction in virus PFU was observed in the stable infected mosquito line with a more than 1.5 log reduction in *w*AlbB-infected mosquito’s bodies (Fig 3A). No infectious virus was detected in saliva from these mosquitoes (Fig 3B).

We observed a small (less than two-fold), non-significant (Fisher exact test, p = 0.13) reduction of virus infection rates between transiently infected and W.T. mosquitoes (Fig 3C). Similarly, a small (less than two-fold), non-significant (Fisher exact test, p = 0.1) reduction in the percentage infectious saliva was found between transiently infected and W.T. mosquitoes (Fig 3D). A significant reduction (Fisher exact test, p = 0.001) in infection rates was observed between the stable *w*AlbB infected mosquito line and W.T. mosquitoes (Fig 3C).

As with the DENV infected mosquitoes, we analysed the *Wolbachia* density in the same transiently and stably infected mosquitoes analysed for WNV (Kunjin strain) replication and transmission. Similar to the DENV infected mosquitoes, our results indicate much lower *Wolbachia* densities in the transiently infected females compared to the stable *w*AlbB infected line (Fig 3E).

### Conclusions

*Wolbachia*, when stably transinfected into mosquito hosts, has been shown to inhibit a range of pathogens, in particular DENV, CHIKV, WNV, YFV and *Plasmodium* [1–8]. There are however, a few studies that have demonstrated infection with *Wolbachia* can lead to enhanced pathogen replication [23, 24, 28, 29]. In particular, a study by Dodson et al. showed that when *w*AlbB transiently infects *C*. *tarsalis*, WNV infection rates can be enhanced [24]. These results are in contrast to a previous study that showed two stable transinfected *Ae*. *aegypti* lines (*w*Mel and *w*MelPop) both inhibited WNV transmission [5].

This would suggest that the interaction between *Wolbachia* and a particular pathogen is highly dependent on either the infection model, the *Wolbachia* strain or the host background. To determine whether the results obtained by Dodson et al. [24] could be a result of the *Wolbachia* infection model, we compared DENV and WNV (Kunjin strain) infection in both *Ae*. *aegypti* transiently infected with *w*AlbB as well as *Ae*. *aegypti* stably transinfected with *w*AlbB. In our experimental setup, DENV and WNV (Kunjin strain) replication was significantly reduced in both *Wolbachia* infection models. In addition, DENV infection rate and transmission rate was significantly reduced in both models. We also observed a small, but not significant reduction in WNV (Kunjin strain) infection and transmission rates in transiently infected mosquitoes.

These observations differ markedly from those described by Dodson et al. [24] and suggest that in *Ae*. *aegypti* mosquitoes, transient and stable *Wolbachia* infections have similar pathogen modulation effects. In *Ae*. *aegypti*, unlike the observations in *C*. *tarsalis*, transient infection with *w*AlbB led to lower virus transmission rates in transiently infected mosquitoes compared to *Wolbachia* naive wild type mosquitoes. We also observed decreased pathogen blocking in transient *Wolbachia* infections compared to stable *Wolbachia* infections. Results generated through the use of transient *Wolbachia* infection models should therefore be interpreted with caution, as they could potentially underestimate the degree of pathogen blocking compared to the stably infected systems typically used for field disease control programs. Most importantly, our results conclusively show no enhancement of either DENV or WNV (Kunjin strain) infection in *Wolbachia* infected *Ae*. *aegypti*.

## Materials and Methods

### Ethics statement

Blood feeding by volunteers (Monash University human ethics permit no CF11/0766-2011000387) for this study was approved by the Monash University Human Research Ethics Committee (MUHREC). All adult volunteers provided informed written consent; no child participants were involved in the study.

### Experimental design

The experimental design for this study is summarised in S1 Fig. We compared the replication and transmission of DENV and WNV (Kunjin strain) in Townsville wild type (W.T.) mosquitoes, W.T. mosquitoes injected with *w*AlbB and the stable *w*AlbB line described in [9]. To generate transient *Wolbachia* infections in female W.T. mosquitoes, *Wolbachia* was isolated from 200 *w*AlbB-infected ovaries and injected into 100 W.T. mosquitoes. For the W.T. and stable *w*AlbB controls, an extraction was done from 200 W.T. ovaries in the same fashion as the *Wolbachia* extraction.

This extract was used to inject 50 W.T. females and 50 *w*AlbB stable infected females. The injected females were incubated for 7 days as described and subsequently allowed to feed on virus infected blood. Fed females were separated from unfed females 24 h post feeding. Females that showed no evidence of feeding were used to analyse the *Wolbachia* infection 7 days post injection, using qPCR and FISH. Engorged females were incubated for a further 7 days. Seven days post feeding, saliva and carcasses (legs and wings were removed) were collected from all fed mosquitoes and assayed for *Wolbachia* density, DENV and WNV (Kunjin strain).

### Mosquito colonies and lines

*Wolbachia*-uninfected *Ae*. *aegypti* eggs were collected from Townsville (Queensland, Australia) in 2015. The *Wolbachia*-infected *w*AlbB mosquito line has been described previously [9] and was a gift from Prof Zhiyong Xi (Michigan State University). All *Ae*. *aegypti* mosquitoes were reared and maintained as described in [6] with the following modification. For hatching, eggs were placed in hatching water (distilled H_2_O, boiled and supplemented with 50 mg/L fish food [Tetramin]) and allowed to hatch for 24 h. Larvae were subsequently reared at a set density of ~150 in 3 L of distilled water as described in [6]. *w*AlbB infected females were backcrossed for 2 generations with W.T. males prior to infection experiments.

### *Wolbachia* isolation for transient infection

*Wolbachia* was isolated from the ovaries of *w*AlbB-infected females according to [24] with the following modifications. Ovaries from 200 *w*AlbB-infected females were dissected on ice and suspended in 50 μL of ice-cold Schneiders media (Sigma-Aldrich) in a 1.5 mL eppendorf tube. The ovaries were crushed briefly using a small plastic pestle after which one 3 mm glass bead was added and the suspension vortexed for 2 min. One mL of ice-cold Schneiders media was added to the homogenised and the solution were centrifuged at 4°C for 5 min at 2000 x g. The supernatant was subsequently sequentially filtered through 5 μM and 1.2 μM syringe filters. The resulting filtrate was centrifuged for 4°C for 10 min at 12000 x g. The supernatant was discarded and the pellet resuspended in 50 μL of ice-cold Schneiders media until use. The extraction was repeated with ovaries from W.T. females for use in control injections. Total bacterial counts were estimated using the LIVE/DEAD staining kit (Thermofisher) and counting the live stained bacteria in a hemocytometer.

### *Generation of transiently Wolbachia*-infected *Ae*. *aegypti*

Female mosquitoes were injected intrathoracically with ~ 1 μL of *Wolbachia* suspension solution (~10^11^ bacteria/ml in Schneiders media) using a pulled glass capillary and a handheld microinjector (Nanoject II, Drummond Sci.). Injected mosquitoes were incubated for 7 days (40 mosquitoes per cup) at 26°C with 65% relative humidity and a 12h light/dark cycle. All injection experiments were conducted in duplicate.

### *Wolbachia* density and distribution

*Wolbachia* density and distribution in the transient infected mosquitoes were compared 7 and 14 days post injection (dpi) to the *w*AlbB line using qPCR and fluorescence *in situ* hybridisation (FISH). DNA was extracted from stable and transiently *Wolbachia* infected mosquitoes using the DNeasy 96 Blood & Tissue kit (Qiagen) according to the manufacturer’s specifications. Quantitative PCR to determine the total relative *Wolbachia* densities of infected lines was performed as described by [30] using primers specific to the gene coding for the *Wolbachia* surface protein (*wsp*) (forward primer 5’-GCATTTGGTTAYAAAATGGACGA-3’, reverse primer 5’- GGAGTGATAGGCATATCTTCAAT-3’), as well as the *Ae*. *aegypti actin* gene (forward primer 5’- GACTACCTGATGAAGATCCTGAC-3’, reverse primer: 5’- GCACAGCTTCTCCTTAATGTCAC-3’) [24]. Statistical differences were determined using a Mann-Whitney (Graphpad Prism version 6.0f).

*Wolbachia* was localized in sections of paraffin-embedded 5–7 day old female mosquitoes by FISH, as described in [31], except that only one probe against 16S rRNA was used and its concentration was increased 10-fold to improve the signal. *w*AlbB was detected using AlbBW5: 5’-CTTAGGCTTGCGCACCTTGCAA-3’, labelled with Alexa 488 dye (green). DAPI was used to stain total DNA.

### Mosquito infection with DENV and the WNV (Kunjin strain)

The propagation and maintenance of dengue virus serotype 2 (DENV-2) ET300 [32] was carried out as previously described [33]. WNV (Kunjin strain) was obtained as a gift from Prof Jason Mackenzie (Melbourne University). WNV (Kunjin strain) was propagated on C6/36 cells in a fashion similar to DENV-2.

Mosquitoes were infected with either DENV-2 (ET300) or WNV (Kunjin strain) (virus strains were grown fresh for each infection) through an infectious blood meal. For feeding experiments with virus infected blood, *Ae*. *aegypti* female mosquitoes were placed in 500 mL plastic containers (40/container), starved for 24 hours and allowed to feed on a 50:50 mixture of defibrinated sheep blood and tissue culture supernatant containing 10^7^ genome copies/mL of DENV-2 or 10^8^ pfu/mL of WNV (Kunjin strain). Feeding was done through a piece of desalted porcine intestine stretched over a water-jacketed membrane feeding apparatus preheated to 37°C for approximately three hours. Fully engorged mosquitoes were placed in 500 mL containers and incubated for 7 days at 26°C with 65% relative humidity and a 12h light/dark cycle. All infection experiments were conducted in duplicate.

### Virus isolation and quantification from infected mosquitoes

Saliva from infected mosquitoes was collected 7 days post feeding (dpf) as described by [26]. Following saliva collection, the bodies of infected mosquitoes were collected in 100 μL serum free RPMI media (Sigma-Aldrich) and stored at -80°C until processing. For DENV-2, the collected saliva was re-injected into 3-day-old W.T. female mosquitoes as described by [26]. Female mosquitoes injected with saliva were incubated for 7 days at 26°C with 65% relative humidity and a 12h light/dark cycle after which they were collected in RPMI media as above. DENV-2 genome copies were subsequently determined in the blood fed and saliva injected mosquitoes using qRT-PCR as described.

For WNV (Kunjin strain), the mosquitoes and saliva were collected as described above. After collection the mosquito bodies were homogenised in a bead beater at 30 beats/min for 3 min using one 3 mm sterile glass bead. The suspension was briefly centrifuged at 2000 x g and 10 μL of the supernatant was used in plaque assays as described by [24]. Collected saliva was used directly in plaque assays.

To quantify DENV-2 genomic copies, total RNA was isolated from DENV-2 injected mosquitoes using the Nucleospin 96 RNA kit (Macherey-Nagel). DENV-2 qPCR analysis was done using cDNA prepared from individual mosquitoes according to [31] using forward primer 5’-AAGGACTAGAGGTTAGAGGAGACCC-3’ and reverse primer 5’-CGTTCTGTGCCTGGAATGATG-3’. Infectious virus titre of WNV (Kunjin strain) was quantified using plaque assays as described by [24].

## Supporting Information

S1 Fig Experimental design followed in this study. Abbreviations used: BF–Blood fed; dpi–days post injection; dpf–days post feeding; W.T.–wild type *Aedes aegypti* females.
(TIF)

S2 Fig DENV infection, replication and transmission in wild type (W.T.), transiently infected (*w*AlbB injected) and the stable transinfected (*w*AlbB) lines 7 days post an infectious blood meal–repeat one. Statistical significance was determined using a Mann-Whitney test (A and D) or a Fisher exact test (B and C). In A and D the mean and error of the mean is indicated. In B and C, the error bars represent 95% confidence levels. A) DENV genome copies in whole mosquito bodies (****, p < 0.0001; **, p = 0.004; Mann-Whitney). B) DENV infection rate as determined by the percentage of individuals infected 7 days post an infectious blood meal (****, p = 0.001; *, p < 0.05; Fisher exact test). C) DENV transmission rate as determined by the percentage of infectious saliva expectorated 7 days post an infectious blood meal, (***, p = 0.0002; *, p = 0.03; Fisher exact test). D) *Wolbachia* density 7 days post an infectious blood meal in transiently infected and the stable transinfected line (****, p < 0.0001; Mann-Whitney).
(TIF)

S3 Fig WNV (Kunjin strain) infection, replication and transmission in wild type (W.T.), transiently infected (*w*AlbB injected) and the stable transinfected (*w*AlbB) lines 7 days post an infectious blood meal–repeat one. Statistical significance was determined using a Mann-Whitney test (A, B and E) or a Fisher exact test (C and D). In A, B and E, the mean and error of the mean is indicated. In C and D, the error bars represent 95% confidence levels. A) WNV (Kunjin strain) PFU per ml in whole mosquito bodies, (**, p < 0.01; Mann-Whitney). B) WNV (Kunjin strain) PFU per ml in saliva (**, p = 0.002; Mann-Whitney). C) WNV (Kunjin strain) infection rate as determined by the percentage of individuals infected 7 days post an infectious blood meal, (*, p = 0.02; Fisher exact test). D) WNV (Kunjin strain) transmission rate as determined by the percentage of infectious saliva expectorated infected 7 days post an infectious blood meal (**, p = 0.002, Mann-Whitney). E) *Wolbachia* density 7 days post an infectious blood meal in transiently infected and the stable transinfected lines (****, p < 0.0001; Mann-Whitney).
(TIF)

S4 Fig DENV infection, replication and transmission in wild type (W.T.), transiently infected (*w*AlbB injected) and the stable transinfected (*w*AlbB) lines 7 days post an infectious blood meal–repeat two. Statistical significance was determined using a Mann-Whitney test (A and D) or a Fisher exact test (B and C). In A and D, the mean and error of the mean is indicated. In B and C, the error bars represent 95% confidence levels. A) DENV genome copies in whole mosquito bodies (***, p ≤ 0.001; **, p = 0.004; Mann-Whitney). B) DENV infection rate as determined by the percentage of individuals infected 7 days post an infectious blood meal, (****, p = 0.0003; Fisher exact test). C) DENV transmission rate as determined by the percentage of infectious saliva expectorated 7 days post an infectious blood meal, (****, p = 0.0002; *, p = 0.03; Fisher exact test). D) *Wolbachia* density 7 days post an infectious blood meal in transiently infected and the stable transinfected line (****, p < 0.0001; Mann-Whitney).
(TIF)

S5 Fig WNV (Kunjin strain) infection, replication and transmission in wild type (W.T.), transiently infected (*w*AlbB injected) and the stable transinfected (*w*AlbB) lines 7 days post an infectious blood meal–repeat two. Statistical significance was determined using a Mann-Whitney test (A, B and E) or a Fisher exact test (C and D). In A, B and E, the mean and error of the mean is indicated. In C and D, the error bars represent 95% confidence levels. A) WNV (Kunjin strain) PFU per ml in whole mosquito bodies, (**, p < 0.006; *, p = 0.04; Mann-Whitney). B) WNV (Kunjin strain) PFU per ml in saliva (****, p < 0.0001, **, p < 0.009; Mann-Whitney). C) WNV (Kunjin strain) infection rate as determined by the percentage of individuals infected 7 days post an infectious blood meal, (**, p = 0.002; Fisher exact test). D) WNV (Kunjin strain) transmission rate as determined by the percentage of infectious saliva expectorated infected 7 days post an infectious blood meal (**, p = 0.007; Mann-Whitney). E) *Wolbachia* density 7 days post an infectious blood meal in transiently infected and the stable transinfected lines (****, p < 0.0001; Mann-Whitney).
(TIF)
