# Supplementary figures and images for: Comparison of Stable and Transient Wolbachia Infection Models in Aedes aegypti to Block Dengue and West Nile Viruses
Source: PLoS Negl Trop Dis. 2017 Jan 4;11(1):e0005275. doi: 10.1371/journal.pntd.0005275 (PMC5241016; doi:10.1371/journal.pntd.0005275)

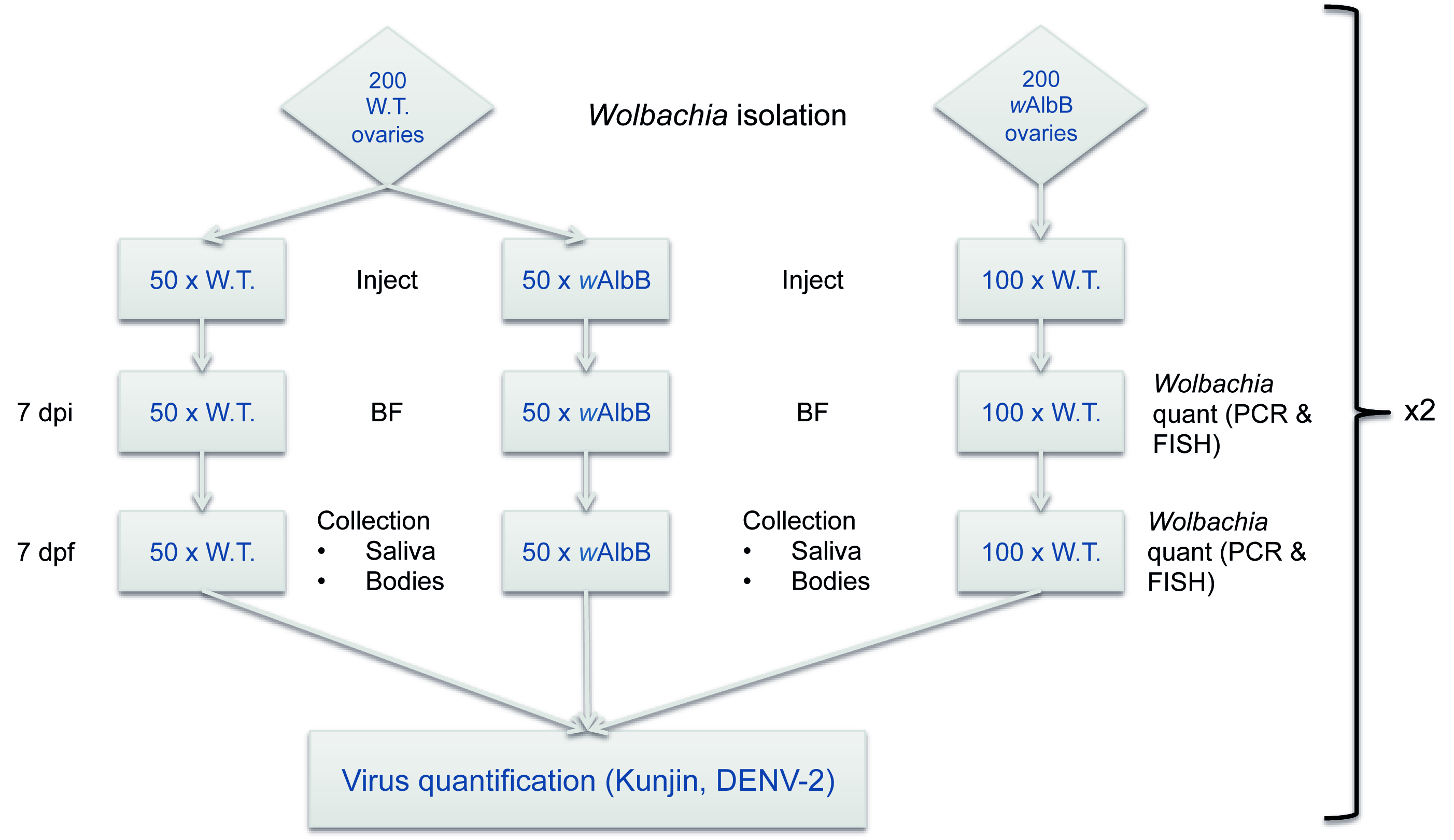

Supplement: S1 Fig — Abbreviations used: BF–Blood fed; dpi–days post injection; dpf–days post feeding; W.T.–wild type Aedes aegypti females. (TIF) [file pntd.0005275.s001.tif]

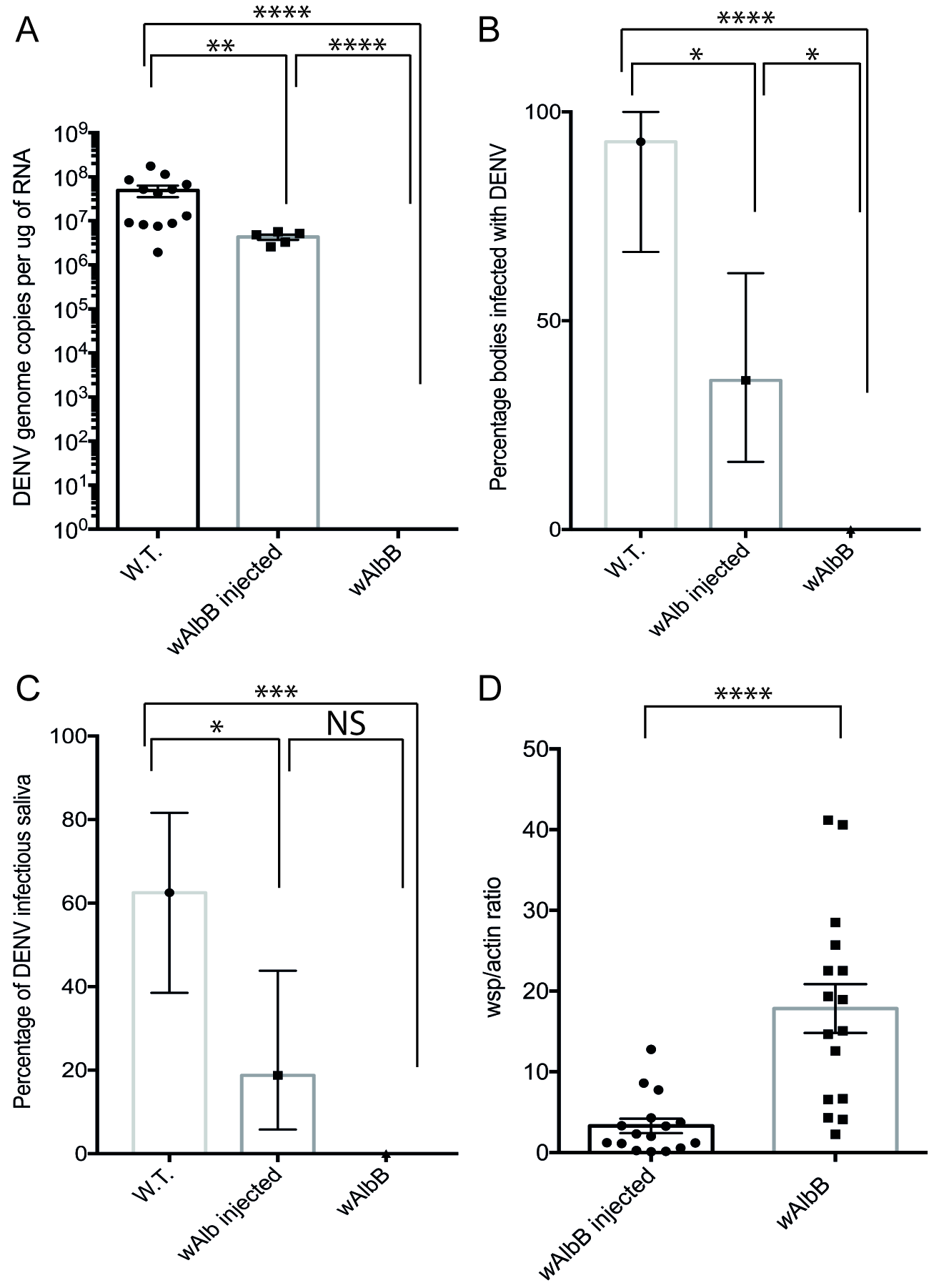

Supplement: S2 Fig — Statistical significance was determined using a Mann-Whitney test (A and D) or a Fisher exact test (B and C). In A and D the mean and error of the mean is indicated. In B and C, the error bars represent 95% confidence levels. A) DENV genome copies in whole mosquito bodies (****, p < 0.0001; **, p = 0.004; Mann-Whitney). B) DENV infection rate as determined by the percentage of individuals infected 7 days post an infectious blood meal (****, p = 0.001; *, p < 0.05; Fisher exact test). C) DENV transmission rate as determined by the percentage of infectious saliva expectorated 7 days post an infectious blood meal, (***, p = 0.0002; *, p = 0.03; Fisher exact test). D) Wolbachia density 7 days post an infectious blood meal in transiently infected and the stable transinfected line (****, p < 0.0001; Mann-Whitney). (TIF) [file pntd.0005275.s002.tif]

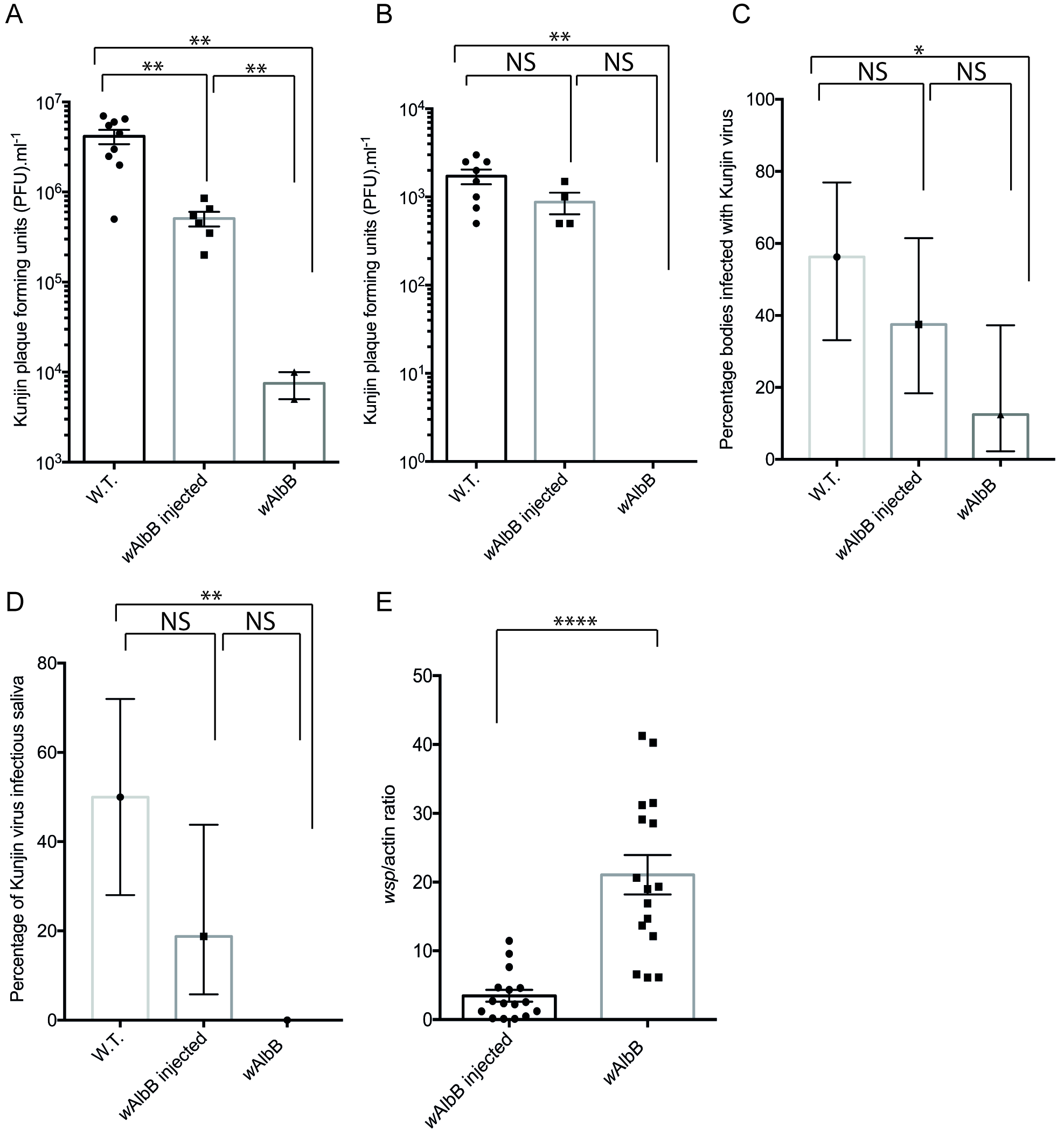

Supplement: S3 Fig — Statistical significance was determined using a Mann-Whitney test (A, B and E) or a Fisher exact test (C and D). In A, B and E, the mean and error of the mean is indicated. In C and D, the error bars represent 95% confidence levels. A) WNV (Kunjin strain) PFU per ml in whole mosquito bodies, (**, p < 0.01; Mann-Whitney). B) WNV (Kunjin strain) PFU per ml in saliva (**, p = 0.002; Mann-Whitney). C) WNV (Kunjin strain) infection rate as determined by the percentage of individuals infected 7 days post an infectious blood meal, (*, p = 0.02; Fisher exact test). D) WNV (Kunjin strain) transmission rate as determined by the percentage of infectious saliva expectorated infected 7 days post an infectious blood meal (**, p = 0.002, Mann-Whitney). E) Wolbachia density 7 days post an infectious blood meal in transiently infected and the stable transinfected lines (****, p < 0.0001; Mann-Whitney). (TIF) [file pntd.0005275.s003.tif]

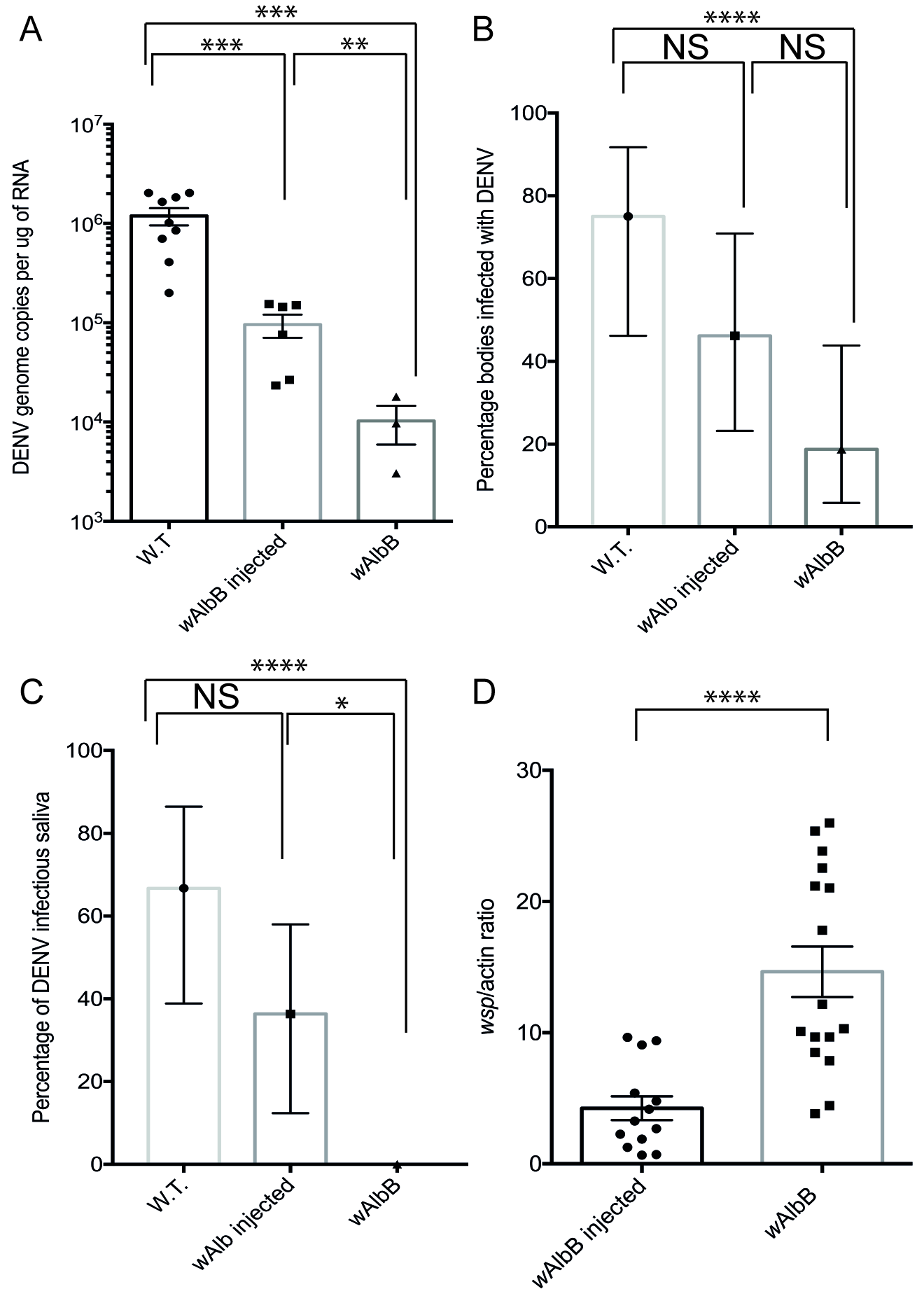

Supplement: S4 Fig — Statistical significance was determined using a Mann-Whitney test (A and D) or a Fisher exact test (B and C). In A and D, the mean and error of the mean is indicated. In B and C, the error bars represent 95% confidence levels. A) DENV genome copies in whole mosquito bodies (***, p ≤ 0.001; **, p = 0.004; Mann-Whitney). B) DENV infection rate as determined by the percentage of individuals infected 7 days post an infectious blood meal, (****, p = 0.0003; Fisher exact test). C) DENV transmission rate as determined by the percentage of infectious saliva expectorated 7 days post an infectious blood meal, (****, p = 0.0002; *, p = 0.03; Fisher exact test). D) Wolbachia density 7 days post an infectious blood meal in transiently infected and the stable transinfected line (****, p < 0.0001; Mann-Whitney). (TIF) [file pntd.0005275.s004.tif]

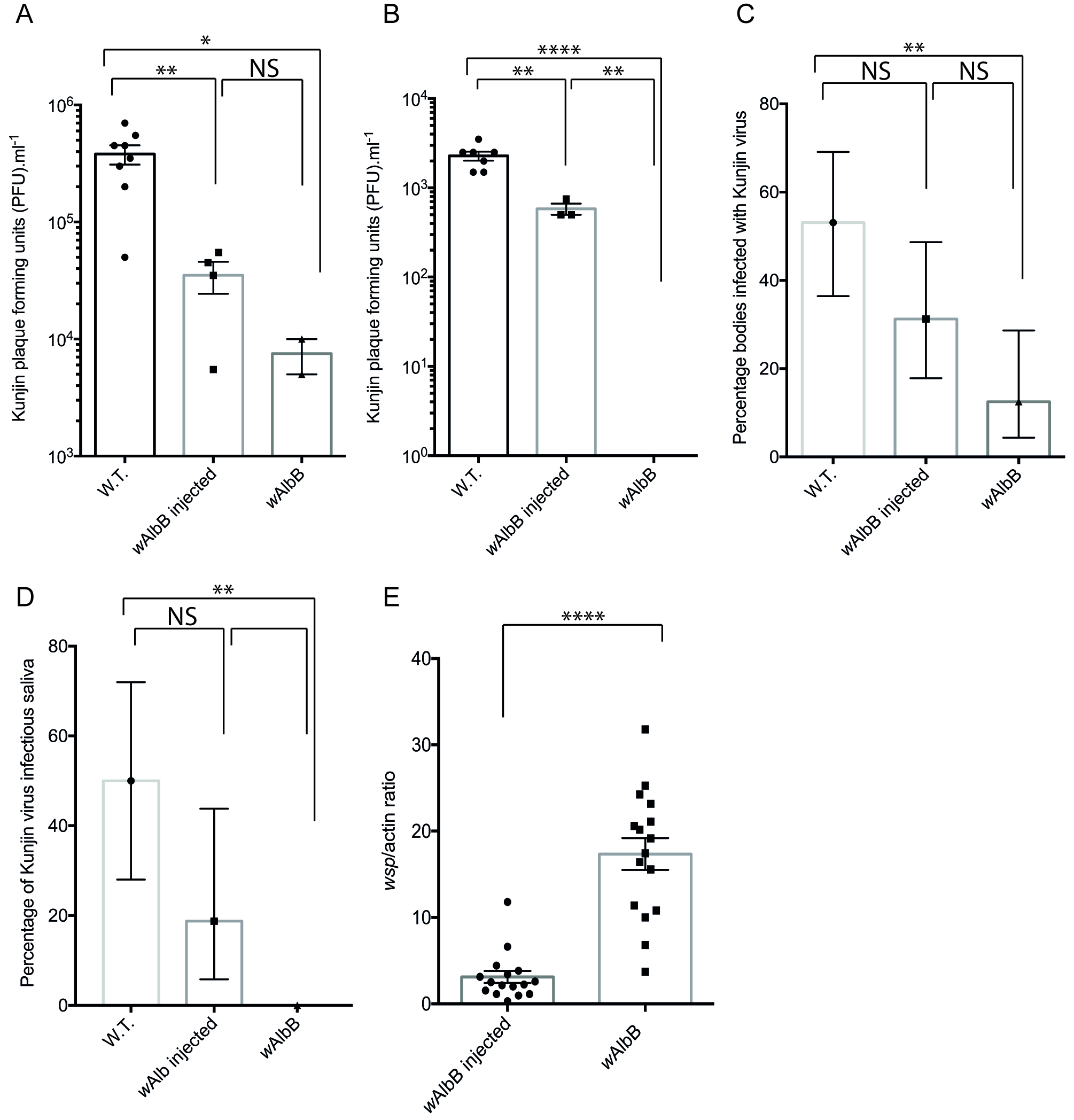

Supplement: S5 Fig — Statistical significance was determined using a Mann-Whitney test (A, B and E) or a Fisher exact test (C and D). In A, B and E, the mean and error of the mean is indicated. In C and D, the error bars represent 95% confidence levels. A) WNV (Kunjin strain) PFU per ml in whole mosquito bodies, (**, p < 0.006; *, p = 0.04; Mann-Whitney). B) WNV (Kunjin strain) PFU per ml in saliva (****, p < 0.0001, **, p < 0.009; Mann-Whitney). C) WNV (Kunjin strain) infection rate as determined by the percentage of individuals infected 7 days post an infectious blood meal, (**, p = 0.002; Fisher exact test). D) WNV (Kunjin strain) transmission rate as determined by the percentage of infectious saliva expectorated infected 7 days post an infectious blood meal (**, p = 0.007; Mann-Whitney). E) Wolbachia density 7 days post an infectious blood meal in transiently infected and the stable transinfected lines (****, p < 0.0001; Mann-Whitney). (TIF) [file pntd.0005275.s005.tif]
